# Differences of Four Work-Related Behavior and Experience Patterns in Work Ability and Other Work-Related Perceptions in a Finance Company

**DOI:** 10.3390/ijerph15071521

**Published:** 2018-07-18

**Authors:** Jan-Bennet Voltmer, Edgar Voltmer, Jürgen Deller

**Affiliations:** 1 Leuphana University of Lüneburg, Institute of Management & Organization (IMO), Universitätsallee 1, 21335 Lüneburg, Germany; deller@leuphana.de; 2 FernUniversität Hagen, Institute of Psychology, Universitätsstraße 47, 58097 Hagen, Germany; 3 Friedensau Adventist University, An der Ihle 19, 39291 Möckern-Friedensau, Germany; edgar.voltmer@thh-friedensau.de; 4 Silver Workers Research Institute (SWRI), Ernst-Reuter-Platz 10, 10587 Berlin, Germany

**Keywords:** behavior and experience patterns, job satisfaction, non-helping profession, presenteeism, work ability, work engagement

## Abstract

The present study applies a salutogenetic approach to psycho-social stress and wellbeing at work and for the first time analyzes the relation of an extended model of four work-related behavior and experience patterns to work related perceptions, like work ability, job satisfaction and turnover intention, or engagement. Employees of an international financial services company (*N* = 182) completed the questionnaire *Work-related behavior and experience pattern* (*Arbeitsbezogenes Verhaltens- und Erlebensmuster*; AVEM). The AVEM has oftentimes been used for research in helping professions, but research in non-helping professions is scarce. In addition to the AVEM, measures of job satisfaction, work ability, work engagement, presenteeism, and turnover intention were included in this study. Almost half (46.2%) of the sample showed a rather unambitious attitude towards work, followed by a burnout-related risk pattern (22.0%), a healthy pattern (19.8%), and a pattern at risk for overexertion (12.1%). Significantly more favorable scores were found for all work-related perceptions in participants with the healthy pattern compared to those with the burnout-related risk pattern, except for turnover intention where no significant differences were found. For work ability and vigor, those with a healthy pattern also had significantly higher scores than those with an unambitious pattern and a pattern at risk for overexertion. Being at risk for burnout not only affects job-related wellbeing and coping resources, but also work ability and work engagement. A need for personnel and organizational development and health promotion is indicated by a high number of individuals with reduced working motivation and risk patterns for overexertion or burnout.

## 1. Introduction

One major threat for the workforce in industrial countries is the increasing number of employees suffering from mental health symptoms. In 2017, mental illness was the second most common reason for absence from work due to illness [1], and the primary cause for early retirement [2]. In the face of a shrinking workforce and the so-called “war for talent” [3,4], the maintenance and improvement of the workforce’s health and wellbeing has become an important topic. The continuing increase in life expectancy, especially in western countries [5], in conjunction with a stagnation or at least slower increase in retirement age [6] has fueled efforts to increase work force participation of older workers [7,8], via maintenance and promotion of health and work ability throughout the (work) lifespan [9].

For that reason, the present study employs a salutogenetic approach to evaluate the relation of a comprehensive set of personal resources as measured by the questionnaire on work-related behavior and experience patterns (AVEM) [10]. Based on the Job Demands–Resources Model (JDR) [11], and the Conservation of Resources Theory (COR) [12], we investigate how the AVEM patterns relate to work ability as a valuable predictor of workforce participation [13,14,15,16]. The JDR employs job resources and job demands to predict burnout and engagement [17]. In the past, the JDR stimulated research on several antecedents and outcomes of occupational health and wellbeing [11]. However, the integration of personal resources, like the subscales and domains of the AVEM, into the model remains an unanswered question with different approaches [18]. According to the COR, the resources from one domain can “spillover” to another domain [12]. Consequently, the personal resources as assessed by the AVEM should be positively related to work ability and the other work-related outcomes. Therefore, we seek to investigate the relation between the four different health patterns resulting from different AVEM scores, and employees’ work ability and other work-related outcomes such as job engagement, job satisfaction, presenteeism, and turnover intention.

### 1.1. Work-Related Behavior and Experience Patterns: The AVEM

The AVEM considers employees’ health as a continuum between health and disease depending on their perception and interaction with the working environment. The salutogenetic approach of the AVEM primarily evaluates factors maintaining and improving health, in contrast to the pathogenetic approach which focuses on the prevention of damages and losses [19,20,21,22]. Based on concepts of health psychology, the AVEM comprises eleven health relevant behaviors and support factors from the following three different domains: (1) professional commitment; (2) resistance against stress; and (3) emotional wellbeing. The underlying concepts of the three domains of the AVEM are the following:

For the domain of professional commitment of the AVEM, (1) the intrinsic part of the effort–reward imbalance model called overcommitment [23,24] and (2) considerations on engagement. Overcommitment is characterized by maladaptive coping with demands, the inability to distance oneself from work, and an extremely ambitious behavior that tends to do more of the same in situations of stress thereby exhausting one’s resources. Overcommitment could therefore intensify the feeling of an effort–reward imbalance that has been shown to possibly result in physical and mental health symptoms [25,26]. Other research demonstrates that the ability to distance oneself from work physically as well as mentally is crucial for health, wellbeing, and the prevention of burnout [27,28]. The conceptualization of and research on engagement, on the other hand, has been fueled by research on its opposite, that is, burnout [29]. Engagement has later been defined as “...a positive, fulfilling, work-related state of mind that is characterized by vigor, dedication, and absorption” [30] and will be described in more detail below.

For the domain of resistance toward stress of the AVEM, the dimensions of offensive coping with stress and resignation tendencies reflect the strategies of problem-oriented and emotional coping of Lazarus’ and Folkman’s transactional model of coping with stress [31]. Supporting this domain are also the models of self-efficacy [32] as well as manageability as one of three factors of the sense of coherence in Antonovsky’s concept of salutogenesis [33].

Lastly, regarding the emotional wellbeing domain, social support, in particular, has gained considerable interest over the last decade as a means of coping and as a valuable resource for health, wellbeing, and the prevention of burnout [31,34,35].

From these domains, four distinct patterns of an individual’s behavioral responses to occupational stress can be derived: in addition to a burnout-related pattern, the AVEM yields a healthy pattern, an unambitious pattern, and a pattern of overexertion, which will be described in more detail in the method section. It has been shown that a typical constellation of personality factors may be more reliable than a single factor alone in predicting health risks [36,37,38,39]. Accordingly, the configuration characteristics across the eleven dimensions of the AVEM are viewed as more informative than the scores of the single dimensions alone. For example, high scores in perfectionism may not be a great health risk per se. However, if combined with a great tendency to exert, a low ability to distance oneself from work, and low social support, it may characterize a severe risk constellation [40].

### 1.2. Work-Related Behavior and Experience Patterns and Work Ability

What work ability is and how it should be assessed is subject to active research [41,42]. Originally, the term was coined to describe the capability of a worker “at present and in the near future […] to do his or her work with respect to work demands, health and mental resources” [43]. Work ability depends on personal physical and mental health conditions in relation to the demands of the individual’s current employment [44]. Lower work ability is associated, for example, with older age, obesity, and lower physical fitness [45]. It is also related to reduced productivity at work [46], increased sickness absence [47], and early retirement [48]. The aspects of work ability are usually assessed using the Work Ability Index (WAI) [44]. It comprises self-perceptions about current work ability in relation to a lifetime best as well as to physical and mental demands, the number of current illnesses, and the estimated work impairment that may be caused by these diseases. Furthermore, it employs the times of sick leave and the expected work ability two years in the future as well as the mental resources of work (satisfaction with daily tasks, activities, and life spirit).

To overcome certain shortcomings of the WAI (e.g., the impact of the psychosocial factors of work on perceived work ability), the Work Ability Survey-R (WAS-R) was developed. The WAS-R is based on the holistic model of work ability, in which work ability is the result of four factors: individual characteristics of the workers, characteristics of the workplace, the social environment of the worker, and society [41,49,50,51]. The model tries to integrate all facets of work ability, instead of only depicting separate factors: “[…] each dimension is examined on the basis of the relation or tension between individuals’ resources and work. At the same time, this model attempts to also take into consideration the contexts in which decisions concerning work, equipment, and the work organization are made” [49]. To assess these factors, the WAS-R contains nine subscales covering individual and organizational capacity [41,51,52]. Individual capacity is measured using subscales on psychological wellbeing, work/life balance, physical health, work benefits, and social support. Organizational capacity is measured using subscales on different characteristics of the supervisor (e.g., social support, discrimination, respect, and autonomy at work). To our knowledge, this is the first study to show how patterns or types of work-related behavior that characterize health-relevant risk factors and coping resources correlate to a perceived ability to work. Since work ability has repeatedly been shown to be related to work force participation [13,14,15,16], this study can provide insight into possible predictive (criterion) validity of the resources and behavior patterns represented by the AVEM regarding later work force participation.

### 1.3. Work-Related Behavior and Experience Patterns and Job Engagement, Job Satisfaction, Presenteeism, and Turnover Intention

In addition to work ability, job satisfaction, turnover intention, work engagement, and presenteeism are important constructs in evaluating an individual’s attachment to and willingness to remain in their job. Job satisfaction is among the most widely studied constructs in occupational psychology [53]. On a very broad level, job satisfaction can be understood as “the pleasurable emotional state resulting from the appraisal of one’s job or job experiences” [54]. In more detail, job satisfaction appears to have multiple facets, like pay, promotions, coworkers, supervision, and the work itself, and involving both cognitive and affective appraisals of these features [53]. Job satisfaction has been found to be related to general life satisfaction, performance (e.g., organizational citizenship behavior), and withdrawal behavior [53]. Low job satisfaction was often found to correlate to high turnover intention [55,56]. Turnover intention has been found to be moderately related to actual turnover [55].

Work engagement is defined as a positive work-related state of mind [29], characterized by vigor, dedication, and absorption, and has been described as a positive antipode to burnout [57,58]. Vigor and dedication were perceived as the direct opposites of exhaustion and cynicism, the core symptoms of burnout [57]. In addition, along with absorption, a key characteristic of the flow state is integrated [59]. Flow characterizes a state of mind that is highly motivated, willing to exert, and correlated to optimal performance [60,61].

In turn, presenteeism is defined as a state when an employee feels so ill that, from their perspective, sick leave would have been reasonable, but they attend work despite this. Ironically, presenteeism has been related to increased subsequent sick leave [62,63]. In a sample of physicians, those with lower job satisfaction had higher scores in presenteeism [64].

To our knowledge, the present study is the first to evaluate the differences between the four work-related behavior and experience patterns and their relation to the personal resources and work-related outcomes described above. Moreover, to date, most studies using the AVEM have focused on the helping professions, including physicians, pastors, and teachers, finding small proportions of the healthy pattern, and high proportions of the burnout-related and unambitious patterns [40,65,66]. Constant contact with suffering individuals, low income, and low prestige are perceived as possible reasons for this distribution of patterns [67]. It contrasted with the results of the few studies addressing the non-helping professionals, such as musicians or entrepreneurs [68,69]. Particularly in the latter, much higher proportions of the healthy pattern and the pattern for overexertion, and much smaller proportions for burnout were found. In the present study, we investigate the distribution of AVEM patterns in a financial services company, where the working environment clearly differs from those of physicians, pastors, teacher, but also from those of musicians and entrepreneurs. Based on the reviewed research above we developed the following hypotheses:

**Hypothesis** **1.** *Specifics of the non-helping professions, including the working conditions in insurance companies, are different from those in helping professions. We therefore hypothesize that, in the insurance employees, a high proportion will be seen with a healthy pattern and a low proportion with a burnout-related pattern.*

**Hypothesis** **2.** *Following research on the JDR Model and employing the holistic model of work ability [45] the personal resources as measured by the AVEM should be positively related to work ability, as well as to job satisfaction and work engagement. In pattern terms: those employees with the healthy pattern should report higher work ability scores, job satisfaction, and work engagement than those with a burnout-related pattern.*

**Hypothesis** **3.** *Furthermore, we hypothesize that turnover intention and presenteeism could be higher in the risk patterns of overexertion and burnout than in the healthy pattern, as presenteeism has been shown to be positively related to burnout [70].*

## 2. Materials and Methods

### 2.1. Participants

Employees of an international financial services company’s office were invited to participate in an online survey on work ability. Of all 580 employees in that office, 406 (70.0%) participated in the questionnaire. Of these, 182 (44.8%) participants additionally completed the AVEM questionnaire, which was clearly marked as an addendum for the participants (hereafter referred to as the AVEM Sample; in contrast, the participants without AVEM data will be referred to as Sample w/o AVEM). Prior to the project, most of the employees had attended oral presentations about the survey on their jour fixes, leading to a relatively high participation rate of over two-thirds in the total sample. Detailed characteristics of the AVEM Sample and the Sample w/o AVEM can be found in Table 1. To estimate generalizability of the AVEM Sample, the characteristics were compared using *χ*²-tests. The corresponding *p*-values can also be found in Table 1.

Both samples consisted of an equal share of female participants, and consisted of individuals of approximately the same age, who had a comparable level of education, and who lived with a partner. In the AVEM sample, the share of participants with leadership responsibility was significantly higher, potentially reflecting an increased interest in the topic of the survey, a perceived role model function, or the increased engagement of this group. Regarding generalizability, however, in absolute numbers, the number of participants with leadership responsibility was small.

### 2.2. Procedure

Upon e-mail invitation, the participants entered a LimeSurvey-based online questionnaire. On the first page, they were welcomed and thanked for their participation, the goals of the study were explained, and informed consent was collected. Participation in the questionnaire was voluntary. Following this introduction, the participants provided some socio-demographic data and answered the scales described subsequently. Upon request of the company, the AVEM was answered last and marked again as a voluntary addendum for the participants. This study was conducted with respect to the ethical guidelines of the Leuphana University Lüneburg, and informed consent of all study participants was ensured.

### 2.3. Measures

#### 2.3.1. Work-Related Behavior and Experience Pattern (AVEM)

The AVEM is a self-report measure for work-related behavioral health risks and resources and coping [10]. These are assessed using eleven subscales on three major domains: professional commitment, resistance towards stress, and emotional wellbeing at work [71]. Scales and example items can be found in Table 2.

Each scale consists of six items. Response options ranged from 1 (“I strongly disagree”) to 5 (“I strongly agree”). Sum scores for the 11 subscales were calculated. These scores were then transformed to norm values. Based on these norm values, the participants were assigned to one of the four patterns [10] that have been externally validated using cluster analysis [72]. A four-cluster solution from the initial sample (*n* = 1598) could be cross-validated in ten boot-strap samples (average κ > 0.80; 71).

Based on discriminant analysis, participants are assigned to the four different clusters by calculating a weighted linear combination of the eleven subscales. Each participant is assigned to one pattern only, with maximum correspondence to his or her individual profile [10,71]. The four profiles can be described as follows [71]:

Pattern G: “Health” (“Gesundheit”). Participants with this pattern have a healthy attitude towards work. They are ambitious but can also distance themselves from work. Resistance to stress and positive emotions are high in these participants [71].

Pattern S: “Unambitious” (“Schonung”). Participants with this pattern have an unambitious attitude towards work. Commitment to work is low in participants with this pattern, while capacity for detachment is high. However, their high tendency to resignation, but beneficial scores in inner balance, satisfaction with life, and the experience of social support reveal the ambivalence in this pattern. Their reduced working motivation could either indicate limited interest in work compared to other areas of life, or rather signal inner frustration with work [71].

Risk pattern A: “Overexertion”. Participants with this pattern are very committed to their work, but face difficulties with emotional distancing from work. Additionally, participants with this pattern have only limited coping capacity in stressful situations and experience increased negative emotions and exhaustion [71].

Risk pattern B: “Burnout”. Participants with this pattern indicate limited professional commitment. They score high on tendencies to resignation and low on emotional distancing and active coping. They experience limited balance and mental stability and limited satisfaction with work and life, and indicate low experience of social support. The core symptoms of burnout can be found in this pattern [10,71].

Research has provided evidence for both construct as well as criterion validity in teaching professions [73], and in rehabilitation contexts [74,75], in part replicating its cluster solution and supporting its relation with work force participation. Additional criterion validity of the AVEM can be drawn from moderate to good correlations of the subscales with measures of related constructs [71], and from correlations of the patterns with emotional stability, mental and physical condition, sickness-related absence, blood pressure, heart rate, type-A behavior, burnout, self-esteem, perceived performance, and exhaustion [76]. Reliability of the subscales in the original study was acceptable to good with values ranging from Cronbach’s α = 0.75 to α = 0.83 [10]. Comparable results were obtained in our samples, with a median Cronbach’s α of α = 0.81 (minimum α = 0.79, maximum α = 0.86).

#### 2.3.2. Work Ability Index (WAI)

The WAI consists of 11 items, rated on different, mostly Likert-type, scales [44]. The total score ranges from 7–49 points, with 7–27 points indicating “critical”, 28–36 “moderate”, 37–43 “good”, and 44–49 “very good” work ability. The questionnaire and interpretation rules can be found online [77,78]. The WAI has been shown to be predictive for work force participation, especially (early) exit from the workforce [15,44,79]. In this study, we used three items of the WAI. The first item on overall work ability was found to be a good estimator on its own of the WAI total score [15]. The other two items were those on diagnosed diseases and the subjective estimation of work impairment due to those diseases [44]. The sum of the three items was calculated. The short scale had a theoretical range of 4–23 points, with higher scores indicating better results. Additional descriptive statistics can be found in Table 3. Considering that the WAI is a formative scale in its nature (hence the “Index” in its name; [80]), internal consistency does not apply to the index itself and its derivatives [81]. However, the WAI has been found to be internally coherent [82], and acceptably test–retest reliable [83].

#### 2.3.3. Work Ability Survey-R (WAS-R)

Complementing the WAI, we used the Work Ability Survey-R (WAS-R) [51]. In contrast to the WAI, the WAS-R measures work ability as the intersection of personal and organizational capacity. Example items are for psychological wellbeing (e.g., “Over the last four weeks have you been able to enjoy your normal day-to-day activities?”), work/life balance (e.g., “Do you feel that your work drains so much of your energy that it has a negative effect on your private life?”), physical health (e.g., “In general, how would you say your health is?”), work benefits (e.g., “Makes me feel good about myself”, prefaced with “What benefits does your work provide for you?”) and social support (e.g., “To what extent can you get help and support from [...]: spouse, friends, relatives?”), characteristics of the supervisor (e.g., social support, “To what extent can you get help and support from [...]: direct supervisor?”, or competence “To what extent do you think your supervisor [...] treats staff as individuals, supports and encourages their development?”), discrimination (e.g., “In the last 12 months have you personally experienced [...] being ignored by colleagues or treated as if you did not exist?”), respect (e.g., “Does management at your workplace respect you?”), and autonomy at work (e.g., “Thinking about your job, are you able to change [...] the order of your tasks?”). In total, the WAS-R consists of 53 items that are primarily rated on 5-point Likert-scales. Scores are calculated by averaging item scores on a subscale level, and then averaging subscale scores. Scores for both personal and organizational capacity, as well as total WAS-R are limited to values between 0 and 100 [41,51]. The relatively high correlation (*r* = 0.50) between the WAS-R and the WAI is an indicator for its validity; however, with 25% shared variance, the WAS-R explains unique variance and is especially useful for practitioners who can derive interventions directly from its broad subscales [41]. The WAS-R has been shown to be adequately correlated with the WAI, but validity with respect to work force participation has yet to be shown [41]. Within this study, we used a modified 52-item version of the WAS-R, omitting one item in the skill usage subscale on self-paid job training that has been found to have only a minimal connection to the scale [41]. The internal consistency in our sample was excellent [84], with Cronbach’s α = 0.94.

#### 2.3.4. Job Satisfaction 

Job satisfaction was assessed using the three items of the Short Form of the Job Diagnostic Survey in their German version [85,86] (example item: “Generally speaking, I am very satisfied with this job”). The total score of job satisfaction was calculated as the sum of the three items [86]. Moderate relations between job satisfaction and job characteristics have been shown in the evaluation of the job characteristics model [87], and job satisfaction has been found to be predictive for turnover intention and withdrawal cognition [55]. The internal consistency was acceptable with Cronbach’s α = 0.75 and close to the α = 0.76 internal consistency of the original study.

#### 2.3.5. Turnover Intention

Turnover intention was measured using two items. One item was adapted from the Job Diagnostic Survey (JDS) [85]: “I oftentimes think of changing jobs”. The other item was adapted from Walsh, Ashford, and Hill [88]: “I have already been looking for other jobs”. Turnover intention was calculated as the sum of the two items. Internal consistency was calculated using the Spearman–Brown formula, since it provides a better fit for two-item measures than does Cronbach’s α [89]. The internal consistency was *r* = 0.87.

#### 2.3.6. Work Engagement

To assess work engagement, we used the short version of the Utrecht Work Engagement Scale (UWES-9) [57]. The scale consists of three subscales on vigor, dedication, and absorption. The sum scores of the three subscales were calculated according to the manual [57]. Some evidence for validity exists regarding its relation to burnout, professional efficacy, and cynicism [57]. Internal consistency was good, with Cronbach’s α = 0.87 for all subscales, and comparable to the original study on the UWES, which yielded internal consistencies between 0.79 and 0.89.

#### 2.3.7. Presenteeism

The subscale on presenteeism from the World Health Organization Health and Performance Questionnaire (HPQ) [90] was used. This subscale consists of three items and allows for the deduction of an absolute presenteeism score, with a theoretical range of 0–100%, and a relative presenteeism score compared to other workers in similar jobs, with a restricted range of 25–200% [90]. Absolute presenteeism is calculated by transforming the item score to percentages. For relative presenteeism, perceived own performance in the last four weeks is divided by the performance of the average employee in that job. Since the scores are calculated as products of the items, no internal consistency is calculated. However, the HPQ has been demonstrated to be highly test–retest reliable [91] and to be closely related to health and productivity losses [92].

## 3. Results

All calculations were conducted with the software R [93]. At first, reversed items were recoded and sum scores for all scales were calculated following the respective manuals or alternative primary sources. Afterwards, descriptive statistics (means, standard deviations, and internal consistencies) for all given subscales were calculated using the pastecs package [94]. To test our hypotheses on the relations between the different constructs, we calculated the intercorrelations of all subscales (see Table 3) using the psych package [95].

The AVEM domain of emotional wellbeing correlated moderately to highly (0.37 < *r* < 0.65) with work ability, job satisfaction, all three facets of work engagement, and absolute presenteeism (see Table 3). These facets of work engagement also correlated highly with work ability as measured by the WAS-R and job satisfaction (0.56 < *r* < 0.69), and moderately with the domains of professional ambition and emotional wellbeing of the AVEM (0.36 < *r* < 0.47). There was a moderate correlation between the two measures of work ability (*r* = 0.50) indicating overlapping but distinct concepts. Two structural equation models were calculated to evaluate the structural model underlying the data: In the first, WAS-R, job satisfaction, UWES: Vigor, UWES: Dedication, UWES: Absorption, and the three AVEM domains Professional Ambition, Resistance toward Stress, and Emotional Wellbeing have been modeled as latent factors of their respective indicators (i.e., WAS-R and the AVEM domains: subscales; job satisfaction and UWES: items). WAI and presenteeism have not been included in the structural model due to their formative nature. Turnover intention has not been included with its two items to allow for identification. In the second model, all indicators of the first model loaded onto a single factor. The first model fit the data better than did the second model (Model 1: *χ*²_(435)_ = 1008.9, *p* < 0.001, Comparative Fit Index (*CFI*) = 0.78, *AIC* = 16,153.2, *BIC* = 16,424.1; Model 2: *χ*²_(464)_ = 1415.0, *p* < 0.001, *CFI* = 0.64, *AIC* = 16,501.3, *BIC* = 16,687.7), yielding more evidence for the distinction of the employed structural model.

### Differences in AVEM Patterns

**Figure 1:**
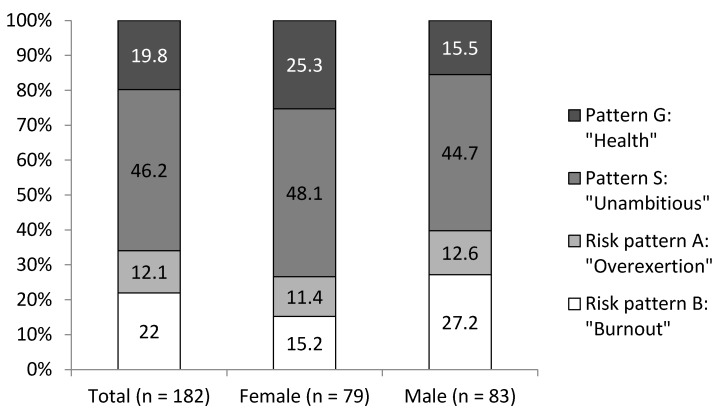
Distribution of work-related behavior and experience patterns in the financial services company’s employees.

**Figure 2:**
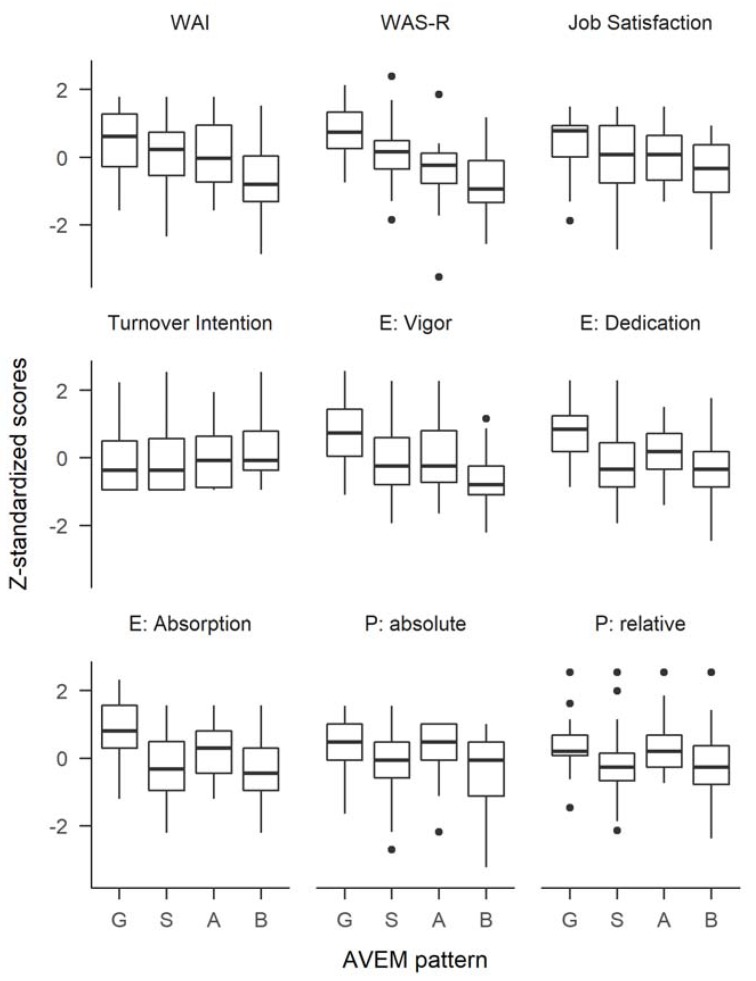
Distribution of other work-related variables within the four work-related behavior and experience patterns G “Health”, S “Unambitious”, A “Overexertion”, and B “Burnout”.

The largest group of participants showed the unambitious pattern S, followed by the burnout-related pattern B, the healthy pattern G, and the pattern A at risk for overexertion (12.1%; Figure 1). To analyze the differences in work ability, job satisfaction, turnover intention, work engagement, and presenteeism between the four patterns of the AVEM, separate between-group one-way analyses of variance (ANOVAs) were conducted. Significant differences were found for work ability in both the WAI (*F*_(3, 178)_ = 7.96, *p* < 0.001) and the WAS-R (*F*_(3, 140)_ = 18.50, *p* < 0.001); job satisfaction (*F*_(3, 178)_ = 6.04, *p* < 0.001); work engagement on all three facets of vigor (*F*_(3, 175)_ = 13.70, *p* < 0.001), dedication (*F*_(3, 170)_ = 11.20, *p* < 0.001), and absorption (*F*_(3, 171)_ = 13.70, *p* < 0.001); and absolute (*F*_(3, 162)_ = 6.81, *p* < 0.001) as well as relative presenteeism (*F*_(3, 159)_ = 4.03, *p* < 0.01). No significant difference was found for turnover intention (*F*_(3, 178)_ = 1.15, *p* = 0.33; see Figure 2). Tukey Honest Significant Differences (HSD) were calculated to further evaluate the differences (see Table 4).

For all measures, except turnover intention, significantly higher—that is, more favorable—scores were found in participants with the healthy pattern G, compared to those with the burnout-related risk pattern B. For the WAS-R and the engagement subscales, those with the healthy pattern G also had significantly higher scores than those with the unambitious pattern S. Regarding the WAS-R and vigor, those with the healthy pattern scored more favorably than those with risk pattern A (overexertion).

## 4. Discussion

In this study, for the first time, we evaluated the differences of four work-related behavior and experience patterns in work ability and other relevant work-related measures in employees of a financial services company. In contrast to a larger number of studies of the helping professions, this study adds to the scarcity of surveys in the non-helping professions. We found a substantial proportion of employees (almost fifty percent) with an unambitious behavior and experience pattern. Those employees with the healthy pattern differed significantly in all other work-related measures (except turnover intention) from those with the pattern at risk for burnout

### 4.1. Hypothesized Differences in Work-Related Behavior and Experience Patterns and Hypothesized Work Outcome Differences

The results of our study differ clearly from other studies examining work-related behavior and experience patterns. Almost a quarter of the participants in our study presented with a burnout-related risk pattern, and almost half with a pattern of reduced working motivation. In contrast, in research among entrepreneurs, less than ten percent presented with these patterns of reduced working motivation or burnout [69]. However, in line with Hypothesis 1, the proportion of the financial services company’s employees with a pattern at risk for burnout was lower, and the proportion with the healthy pattern was higher than in typical helping professions, such as pastors [69] and physicians [68]. The strain of constantly working with ill individuals or clients in need has often been described as a reason for increased burnout rates and decreased health in the helping professions [67,96]. This stress factor does not normally affect employees of a financial services company.

In line with Hypothesis 2, we found those financial services company’s employees with the healthy pattern to report significantly higher scores than those in the burnout pattern for work ability, job satisfaction, and work engagement. These findings are in line with models that emphasize the importance of personal resources and coping behaviors like those measured by the AVEM for health, wellbeing, and the prevention of burnout [11,12]. Additionally, they extend the knowledge about the behavior and experience patterns and demonstrate how deeply and wide-ranging the risk for burnout, including the limitation of personal resources, can be. Recent studies have identified mental symptoms and diseases (including burnout) as the second most cited reason for sick leave and early retirement [1,2]. These may, therefore, not only be threats to individual health but also to productivity and the shrinking workforce.

Regarding Hypothesis 3, no significant differences between the patterns have been found for turnover intention. In line with our hypothesis, those in the healthy pattern yielded more favorable scores in presenteeism compared with those in the unambitious pattern (absolute and relative presenteeism), as well as compared with those in risk pattern B (absolute presenteeism).

### 4.2. Further Results on Work-Related Behavior and Experience Patterns and Work Outcome Differences

Another striking finding of our study was that the proportion of the financial services company’s employees with the unambitious pattern of reduced working motivation was highest of all the above-mentioned professions. Employees with this unambitious pattern scored lower in all three subscales of work engagement than those with a healthy pattern. On the one hand, this may seem natural: one domain of the AVEM is professional ambition, which might be perceived as a related construct of work engagement. Lower scores in this domain are the key characteristic of the unambitious pattern S. Consequently, the finding could be interpreted as an external cross validation. However, the subscale Absorption also represents one of the most central conditions of flow experiences [59]. Flow state describes experiences in leisure or work activities when individuals are highly motivated, acting at ease, and at best performance [60,61]. The significantly lower scores of the unambitious patterns S compared with the healthy pattern G in this subscale may indicate that flow experiences and optimal performance in these employees could be less likely than in those with the healthy pattern G. This finding could be interpreted in a way that pattern S behavior may not be a sound coping mechanism when facing overwhelming work demands. The lower job satisfaction of employees with the unambitious pattern compared with those with the healthy pattern (though not significant) may add to this impression. It has also been shown in longitudinal studies that there was a substantial transition of participants from this unambitious pattern to the burnout-related pattern [71].

The fact that in work ability, measured by the WAS-R, and the Vigor subscale, those employees with a healthy pattern G not only scored significantly higher than those with the burnout-related risk pattern B, but also higher than those with the unambitious pattern S and the pattern A at risk for overexertion, emphasizes that neither an attitude of overexertion nor going easy at work may be related to optimal self-perceived work ability or vigor at work.

### 4.3. Implications for Health Promotion

Since various studies agree that promoting the health behavior of employees and organizational capacity (i.e., the physical and psychosocial work environment) could be beneficial for job satisfaction, working motivation, work engagement, and performance, as well as for the prevention of sick leave, early retirement, and burnout [51,97,98,99,100], the implementation of integrated occupational health management and organizational development projects should be promoted.

Two important indications should be taken from the findings of our study:

1. Different situations, environments, and individuals call for different interventions to promote health and wellbeing at work. In our study, a substantial proportion of participants indicated a rather unambitious attitude towards work or even a burnout-related risk pattern. These individuals are in part strongly differing from those participants with a healthier pattern in terms of perception of and behavior at work. Applying a “one-size-fits-all” intervention will, at a minimum, waste resources on one of the groups but, in the worst case, could also have harmful effects on one of the groups, while being beneficial to the other. Studies show that more tailored approaches could tackle workplace health challenges more effectively [9,101]. Based on results in rehabilitation patients [75] and teachers [102], a health-training program has been developed that addresses the specific needs of the respective risk patterns [102]. Starting with the AVEM as a diagnostic of the four work-related behavior and experience patterns, accompanied by additional general measures of work organization and stress management (e.g., problem-solving training, time- and self-management, training of communication and social competence, goal setting), specific recommendations for clients with risk patterns A and B are made. Both have in common an inability to distance oneself from work and to relax. In clients with risk pattern A, this is mainly self-imposed. There is still energy to change behavior and circumstances. Emphasis on training in self- and time-management as well as relaxation techniques could be helpful. Clients with risk pattern B often feel like victims of circumstances and lack the energy for change. Therefore, emotional stabilization and support in goal setting and proactive coping are needed. For those employees with unambitious pattern S, the main challenge is to foster work motivation and engagement. Measures of human resource development like job enrichment or job enlargement may be appropriate steps to overcome the unambitious attitude.
2. As these recommendations primarily addressed personal behavior, and while addressing only physical health factors can already improve health and wellbeing [101,103,104], a systemic approach that addresses also the organizational and even the societal level (i.e., integrated programs of occupation health management) delivered more promising results [105,106,107,108]. After a diagnostic approach (sick leave analysis, employee survey, work process analysis), these programs usually comprise measures of work process and equipment optimization (e.g., shift schedules, office chair and desk, prohibition of smoking) at the workplace, in addition to measures that address the identified specific health risk behaviors (e.g., stress management training, preventive back and spine exercise courses). Great emphasis must be placed on the participation of employees in the development of measures and an evaluation of the results. Moreover, emphasis should also be placed on the supervisor and leadership style: supervisor behavior not only has a direct impact on health [109], but studies also suggest indirect effects via working conditions and personality of the worker [110], and supervisors not only act as occupational role models, but also as role models on the border between occupational and nonoccupational life [111]. However, according to surveys of the German insurance branch, a preliminary diagnostic step or an evaluation of effects was seldom used, and measures of individual health promotion dominated. Integration in an occupational health and human resource management program increased the likelihood and number of performed measures [112,113]. Within the participating company in our study, the study results must be seen as only the first step to derive and develop measures to tackle occupational health from both an individual perspective and a company perspective. Further research evaluating the impact of organizational culture, supervisor and leadership style, and societal factors is needed.

### 4.4. Limitations

The response rate of this survey was satisfactory. Almost one-third of this office’s workforce completed the AVEM. The sample therefore has a relatively high likelihood to represent this office’s workforce, especially with regard to the mostly nonsignificant differences between the total and the AVEM samples. However, since the AVEM was marked as an addendum for the participants, selection bias of course could have had an impact on the results. No post hoc power analysis was conducted due to recommendations by Hoenig and Heisey [114]. However, since the total sample as well as three of the four pattern groups exceeded 30 participants, the distribution of estimators can be expected to be normal, resulting in reliable estimators [115].

Since the data were drawn from one financial services company, and there were smaller proportions of women and older employees, the generalizability of the results for the general workforce is limited.

Furthermore, cross-sectional data do not allow conclusions regarding cause or development of patterns of work ability. Consequently, it is possible that, for example, higher job satisfaction of those participants with a healthy pattern is not a result of this healthy pattern but is in fact the cause of this healthy pattern or—vice versa—of a pattern at risk for burnout. In that case, individuals with limited job satisfaction would be at risk of developing burnout-related behavior and experience patterns. In line with recent research, we hypothesized the behavior and experience pattern to have an impact on the presented outcome variables [11]. However, only longitudinal studies could provide more evidence for causality and the direction of these relations.

Additionally, only self-report measures were employed in this study, with two important implications: First, the findings here provide an insight into the experience of the participants. Although many items are formulated in a behavior-related way, they could still be biased by erroneous perceptions of the participants [116,117]. However, it has also to be considered that the experience or subjective perception of emotional exhaustion and burnout might be more relevant for the emotional wellbeing and performance of a person than objective estimations of third parties. Second, reliance on only self-report measures additionally increases the risk of common method variance, resulting in possible common method bias [118]. Active debate on the issue of common method bias exists [118,119,120,121], and no definitive advice can be given. In this study, however, only self-report measures were available. To minimize the impact of common method bias, anonymity of the participants’ data was strongly emphasized [118]. To validate our findings, and to account for both common method bias and the reliance on only self-report measures, diverse measures of the respective constructs should be employed in longitudinal or repeated measures designs.

## 5. Conclusions

Being at risk for burnout affects not only job-related wellbeing and coping resources but also work ability, job satisfaction, and work engagement. The issue of reduced working motivation in almost half of the participants, and more than a third of these financial services company’s employees presenting with risk patterns for overexertion or burnout, indicates a need for personnel and organizational development as well as health promotion in this setting.

## Figures and Tables

**Table 1:** Sample characteristics of the financial services company’s employees and *p*-values of the *χ*² difference tests.

Variable	Sample w/o AVEM (*n* = 224)		AVEM Sample (*n* = 182)		*P_χ_*_²_
Gender female *n* (%)	109	49.1	79	43.4	0.254
Age *m* (*SD*)	44.6	10.5	43.9	10.2	0.492
Education *n* (%)	-	-	-	-	0.063
(Intermediate) secondary	76	35.5	56	30.8	-
Higher education entrance	94	43.9	69	37.9	-
Tertiary education	43	20.1	57	31.3	-
PhD	1	0.5	0	0.0	-
Leadership *n* (%)	12	5.6	21	11.7	0.030
Partner *n* (%)	165	73.7	140	76.9	0.450

Note: *m* = mean, *n* = number, *SD* = standard deviation.

**Table 2:** Work-related behavior and experience pattern (AVEM) dimensions with item examples.

AVEM Dimensions	Item Example
1. Subjective significance of work	Work is the most important element in my life
2. Career ambition	I want to achieve more in my career than most people I know
3. Tendency to exert	If necessary, I will work until I am exhausted
4. Striving for perfection	My work should never contain errors or deficiencies
5. Emotional distancing	After work is over I can forget about it quickly
6. Resignation tendencies	I quickly resign myself to lack of success
7. Offensive coping with problems	For me, difficulties are there to overcome
8. Balance and mental stability	I do not get upset easily
9. Satisfaction with work	Until now I have been successful in my work
10. Satisfaction with life	So far, I have been satisfied with my life
11. Experience of social support	My partner shows understanding for my work

**Table 3:** Correlation matrix of the AVEM, WAI, WAS-R, Job Satisfaction, Turnover Intention, the Utrecht Work Engagement Scale (UWES) Vigor, Dedication, and Absorption, as well as absolute and relative Presenteeism with internal consistency—if applicable—in the main diagonal.

	Variable	*n*	*min*	*max*	*m*	*SD*	1	2	3	4	5	6	7	8	9	10	11
1	AVEM Professional Ambition	182	1.2	7.6	4.6	1.0	0.87										
2	AVEM Resistance toward stress	182	1.8	6.8	4.8	0.9	0.32 ***	0.50									
3	AVEM Emotional wellbeing	182	1.0	8.3	4.7	1.3	0.21 **	0.40 ***	0.70								
4	Work ability Index (WAI)	182	5.0	23.0	16.1	3.9	0.17 *	0.32 ***	0.42 ***								
5	Work ability Survey (WAS-R)	144	25.6	94.8	66.9	11.7	0.21 *	0.36 ***	0.65 ***	0.50 ***	0.94						
6	Job Satisfaction	182	6.0	21.0	15.7	3.6	0.26 ***	0.18 *	0.37 ***	0.32 ***	0.57 ***	0.68					
7	Turnover Intention	182	2.0	14.0	5.3	3.4	−0.06	−0.02	−0.20 **	−0.19 *	−0.39 ***	−0.62 ***	0.87				
8	UWES Vigor	179	4.0	21.0	11.9	3.6	0.36 ***	0.29 ***	0.47 ***	0.44 ***	0.69 ***	0.56 ***	−0.34 ***	0.88			
9	UWES Dedication	174	3.0	21.0	12.3	3.8	0.36 ***	0.15	0.46 ***	0.34 ***	0.65 ***	0.62 ***	−0.36 ***	0.80 ***	0.86		
10	UWES Absorption	175	3.0	21.0	11.8	4.0	0.45 ***	0.20 **	0.42 ***	0.33 ***	0.63 ***	0.58 ***	−0.36 ***	0.80 ***	0.86 ***	0.86	
11	Presenteeism—absolute	166	10.0	100.0	71.0	18.8	0.30 ***	0.26 ***	0.38 ***	0.36 ***	0.43 ***	0.37 ***	−0.13	0.43 ***	0.43 ***	0.43 ***	
12	Presenteeism—relative	163	25.0	200.0	109.4	35.6	0.37 ***	0.19 *	0.22 **	0.22 **	0.15	0.22 **	−0.11	0.20 *	0.18 *	0.20 *	0.65 ***

* *p* < 0.05; ** *p* < 0.01; *** *p* < 0.001.

**Table 4:** Differences of work-related behavior and experience patterns (AVEM) in relevant other work-related variables.

Variable	Pattern G “Health”	Pattern S “Unambitious”	Risk Pattern A “Overexertion”	Risk Pattern B “Burnout”	HSD *p*_∆_ < 0.05
*n*	32–36	62–84	18–22	32–40	-
	*m* (*SD*)	*m* (*SD*)	*m* (*SD*)	*m* (*SD*)	-
WAI	17.72 (0.60)	16.44 (0.36)	16.23 (0.84)	13.78 (0.68)	G > B, S > B
WAS-R	75.71 (1.41)	68.31 (1.26)	62.49 (3.01)	57.92 (1.82)	G > S, G > A, G > B, S > B
Job satisfaction	17.39 (0.51)	15.65 (0.39)	15.95 (0.61)	14.05 (0.60)	G > B
Turnover intention	4.97 (0.60)	5.02 (0.36)	5.27 (0.70)	6.17 (0.58)	-
UWES Vigor	14.53 (0.51)	11.67 (0.36)	11.95 (0.79)	9.74 (0.48)	G > S, G > A, G > B, S > B
UWES Dedication	15.11 (0.48)	11.57 (0.42)	12.77 (0.68)	10.84 (0.60)	G > S, G > B
UWES Absorption	15.06 (0.56)	10.91 (0.41)	12.50 (0.78)	10.26 (0.59)	G > S, G > B
P: absolute	81.21 (2.60)	69.74 (2.07)	74.74 (3.77)	62.43 (3.34)	G > S, G > B
P: relative	123.17 (5.30)	103.05 (3.98)	123.19 (8.26)	102.72 (6.29)	G > S

WAI, Work Ability Index; WAS-R, Work Ability Survey-R; UWES Vigor/Dedication/Absorption, Utrecht Work Engagement Subscale Vigor/Dedication/Absorption; P: absolute/relative, Presenteeism absolute/relative; HSD, Tukey Honest Significant Differences. “G > B” indicates a significant difference between participants with pattern G and risk pattern B.
